# Supplementary material for: BMI1-Inhibitor PTC596 in Combination with MCL1 Inhibitor S63845 or MEK Inhibitor Trametinib in the Treatment of Acute Leukemia
Source: Cancers (Basel). 2021 Feb 2;13(3):581. doi: 10.3390/cancers13030581 (PMC7867282; doi:10.3390/cancers13030581)
Supplement: Supplementary file 1 [file cancers-13-00581-s001.pdf]

cancers

*Supplementary materials*

# BMI1-Inhibitor PTC596 in Combination with MCL1 Inhibitor S63845 or MEK Inhibitor Trametinib in the Treatment of Acute Leukemia

**Katja Seipel [1,2], Basil Kopp [1], Ulrike Bacher [3] and Thomas Pabst [2,*]**

[1] Department for Biomedical Research (DBMR), University of Berne, 3008 Bern, Switzerland; katja.seipel@dbmr.unibe.ch or katja.seipel@insel.ch (K.S.); basil.kopp@students.unibe.ch (B.K.)
[2] Department of Medical Oncology, University Hospital Berne, 3010 Bern, Switzerland
[3] Department of Hematology, University Hospital Berne, 3010 Bern, Switzerland; veraulrike.bacher@insel.ch
* Correspondence: thomas.pabst@insel.ch; Tel.: +41-31-632-8430; Fax: +41-31-632-3410

## Supplementary materials

**Citation:** Seipel, K.; Kopp, B.; Bacher, U.; Pabst, T. BMI1-Inhibitor PTC596 in Combination with MCL1 Inhibitor S63845 or MEK Inhibitor Trametinib in the Treatment of Acute Leukemia. *Cancers* **2021**, *13*, 581.https://doi.org/10.3390/cancers13030581

Academic editor: Alberto Maria Martelli
Received: 17 December 2020
Accepted: 28 January 2021
Published: 02 February 2021

**Publisher's Note:** MDPI stays neutral with regard to jurisdictional claims in published maps and institutional affiliations.

**Copyright:** © 2021 by the authors. Submitted for possible open access publication under the terms and conditions of the Creative Commons Attribution (CC BY) license (http://creativecommons.org/licenses/by/4.0/).

A)

| Drug | Dose | Effect |
|---|---|---|
| PTC596 [nM] | 100 | 0.95 |
| | 200 | 0.85 |
| | 400 | 0.68 |
| APR246 [mM] | 10 | 0.98 |
| | 20 | 0.96 |
| S63845 [nM] | 25 | 0.71 |
| | 50 | 0.59 |
| Trametinib [nM] | 10 | 0.97 |
| | 100 | 0.93 |
| PKC412 [nM] | 50 | 0.78 |
| | 100 | 0.73 |
| HDM201 [nM] | 50 | 0.87 |
| | 100 | 0.78 |
| CI [nM] | 50 | 0.71 |
| | 100 | 0.55 |

B)

CI Data for Non-Constant Combo:
PTC596+APR246

| Dose P | Dose A | Effect | CI |
|---|---|---|---|
| 100 | 10 | 0.85 | 0.96 |
| 200 | 10 | 0.72 | 0.95 |
| 400 | 10 | 0.5 | 0.91 |
| 100 | 20 | 0.7 | 0.99 |
| 200 | 20 | 0.62 | 1.08 |
| 400 | 20 | 0.45 | 1.07 |

CI Data for Non-Constant Combo:
PTC596+S63845

| Dose P | Dose S | Effect | CI |
|---|---|---|---|
| 100 | 25 | 0.51 | 0.41 |
| 200 | 25 | 0.44 | 0.43 |
| 400 | 25 | 0.38 | 0.57 |
| 100 | 50 | 0.42 | 0.42 |
| 200 | 50 | 0.35 | 0.39 |
| 400 | 50 | 0.32 | 0.54 |

CI Data for Non-Constant Combo:
PTC596+Trametinib

| Dose P | Dose T | Effect | CI |
|---|---|---|---|
| 100 | 10 | 0.72 | 0.29 |
| 200 | 10 | 0.6 | 0.41 |
| 400 | 10 | 0.42 | 0.51 |
| 100 | 100 | 0.68 | 0.26 |
| 200 | 100 | 0.55 | 0.36 |
| 400 | 100 | 0.4 | 0.49 |

CI Data for Non-Constant Combo:
PTC596+PKC412

| Dose P | Dose PKC | Effect | CI |
|---|---|---|---|
| 100 | 50 | 0.66 | 0.45 |
| 200 | 50 | 0.53 | 0.39 |
| 400 | 50 | 0.39 | 0.49 |
| 100 | 100 | 0.58 | 0.37 |
| 200 | 100 | 0.5 | 0.39 |
| 400 | 100 | 0.3 | 0.38 |

CI Data for Non-Constant Combo:
PTC596+HDM201

| Dose P | Dose HDM | Effect | CI |
|---|---|---|---|
| 100 | 50 | 0.78 | 0.85 |
| 200 | 50 | 0.67 | 0.77 |
| 400 | 50 | 0.49 | 0.74 |
| 100 | 100 | 0.7 | 0.90 |
| 200 | 100 | 0.63 | 0.89 |

CI Data for Non-Constant Combo:
PTC596+CI

| Dose PTC | Dose CI | Effect | CI |
|---|---|---|---|
| 100 | 50 | 0.65 | 1.01 |
| 200 | 50 | 0.62 | 1.11 |
| 400 | 50 | 0.58 | 1.34 |
| 100 | 100 | 0.58 | 1.33 |
| 200 | 100 | 0.56 | 1.41 |
| 400 | 100 | 0.52 | 1.54 |

**Figure S1.** Combination index (CI) MOLM-13. (A) Input of dose-effect values of cell viability in MOLM-13 cells treated with PTC596 and other targeted compounds or conventional induction therapy (CI, nM) and (B) output of combination index values as calculated on CompuSyn software.

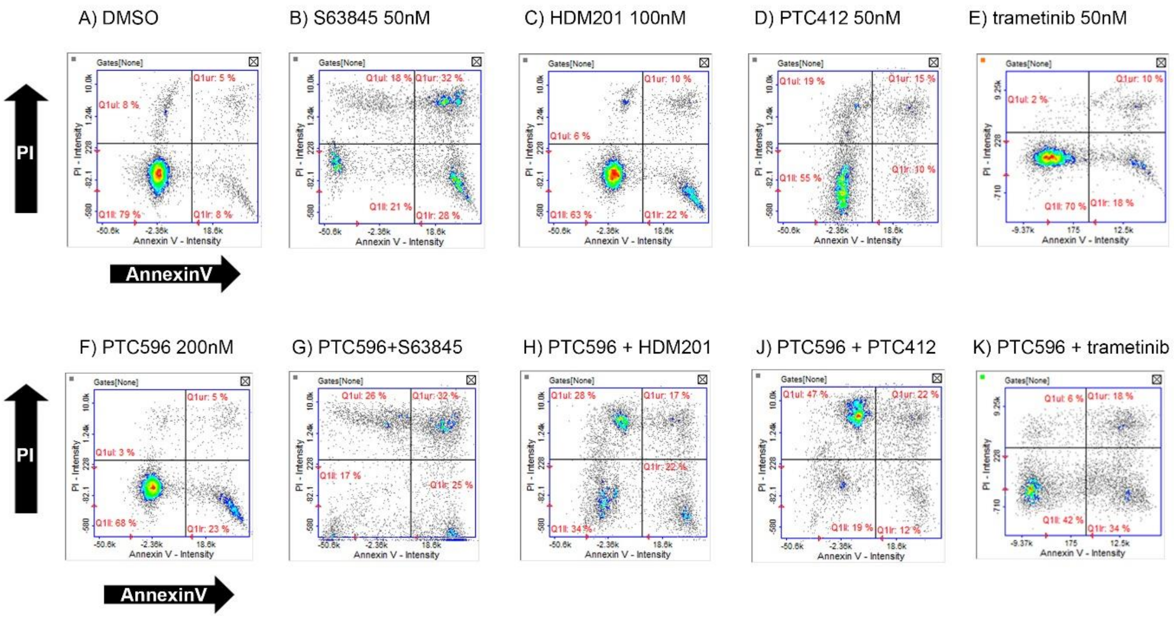


**Figure S2.** Imaging cytometry in MOLM-13 cells treated for 20 hrs with targeted compounds alone A) mock-treated, B) S63845, C) HDM201, D) PTC412, E) trametinib, F) PTC596, G) PTC596 and in combination G) PTC596 and S62834, H) PTC596 and HDM201, J) PTC596 and PTC412, K) PTC596 and trametinib, showing induction of apoptosis and cell death using annexinV and PI staining.

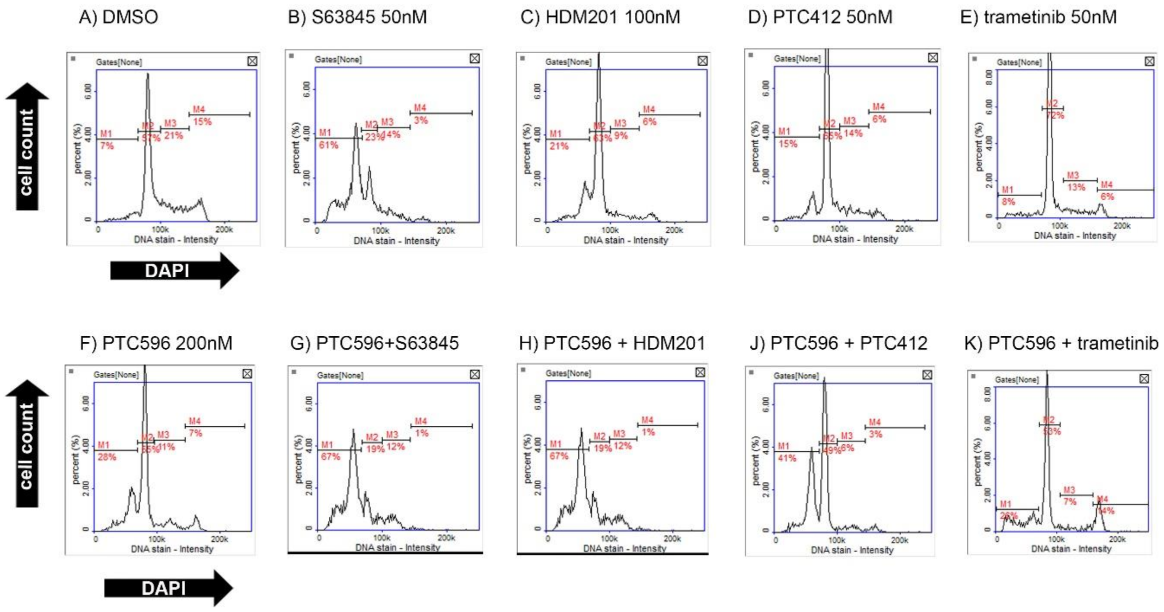


**Figure S3.** Imaging cytometry in MOLM-13 cells treated for 20 hrs with targeted compounds alone A) mock-treated, B) S63845, C) HDM201, D) PTC412, E) trametinib, F) PTC596, G) PTC596 and in combination G) PTC596 and S62834, H) PTC596 and HDM201, J) PTC596 and PTC412, K) PTC596 and trametinib, showing induction of cell cycle arrest and cell death (subG1 fraction) using DAPI staining.
